# Tropomodulin–Tropomyosin Interplay Modulates Interaction Between Cardiac Myosin and Thin Filaments

**DOI:** 10.3390/biom15050727

**Published:** 2025-05-16

**Authors:** Galina V. Kopylova, Anastasia M. Kochurova, Evgeniia A. Beldiia, Andrey V. Slushchev, Victoria V. Nefedova, Natalia S. Ryabkova, Ivan A. Katrukha, Daria S. Yampolskaya, Alexander M. Matyushenko, Daniil V. Shchepkin

**Affiliations:** 1 Institute of Immunology and Physiology of the Russian Academy of Sciences, Yekaterinburg 620049, Russiad.shchepkin@iip.uran.ru (D.V.S.); 2 Research Center of Biotechnology of the Russian Academy of Sciences, Moscow 119071, Russia; slushevav@gmail.com (A.V.S.); victoria.v.nefedova@mail.ru (V.V.N.); daria_logvinova@mail.ru (D.S.Y.); 3 Department of Biochemistry, Faculty of Biology, Lomonosov Moscow State University, Moscow 119991, Russia; n.ryabcova@gmail.com (N.S.R.);; 4 HyTest Ltd., 20520 Turku, Finland

**Keywords:** tropomodulin, cardiac tropomyosin isoforms, actin-associated proteins, cardiac myosin isoforms, actin–myosin interaction, calcium regulation, in vitro motility assay

## Abstract

Tropomodulin (Tmod) is an actin-binding protein that interacts with tropomyosin and the actin filament at the pointed end. The influence of Tmod on the thin filament activation in the myocardium is not clear. We studied the interactions of Tmod1 and Tmod4 with the cardiac tropomyosin isoforms Tpm1.1 and Tpm1.2 using size-exclusion chromatography, a pull-down assay, and cross-linking with glutaraldehyde. We found that Tmod1 and Tmod4 form complexes with both Tpm1.1 and Tpm1.2, indicating durable interactions between these proteins. The effects of both Tmods on the actin–myosin interaction were studied using an in vitro motility assay. Tmod did not affect the sliding velocity of bare F-actin. Tmod1 slightly dose-dependently decreased the sliding velocity of F-actin–Tpm1.1 filaments and had no effect on the velocity of F-actin–Tpm1.2 filaments. With ventricular myosin, Tmod1 reduced the calcium sensitivity of the sliding velocity of thin filaments containing Tpm1.1 but did not affect it with filaments containing Tpm1.2. With atrial myosin, Tmod1 decreased the calcium sensitivity of the sliding velocities of thin filaments containing both Tpm1.1 and Tpm1.2. We can conclude that Tmod takes part in the regulation of actin–myosin interactions in the myocardium through interactions with Tpm. The effect of Tmod on the activation of thin filaments depends on the protein isoforms.

## 1. Introduction

It has been shown that the length of a thin filament in the sarcomere of cardiomyocytes significantly affects the mechanics of the left ventricular myocardium [1]. Dysregulation of thin filament length caused by dysfunction of proteins capping the actin filament contributes to heart failure [1,2,3]. In muscle sarcomere, the length of a thin filament is determined by the dynamics of attachment and detachment of actin monomers at the slow-growing (pointed, minus) end, which are regulated by tropomodulin (Tmod) and leiomodin (Lmod) [4,5,6,7,8]. Tmod binds to the minus end of the actin filament and prevents its disassembly and attachment of new actin monomers [9], while Lmod displaces Tmod and promotes attachment of new actin monomers and elongation of the actin filament [5,10]. In striated muscle sarcomere, two Tmod isoforms are present. Tmod1, encoded by the *TMOD1* gene, is expressed in both cardiac and skeletal muscles, while Tmod4 (*TMOD4* gene) is found only in skeletal muscles [11,12]. Also, in the actin cytoskeleton that is not part of striated muscle sarcomere, Tmod3 (*TMOD3* gene) is expressed [12].

Tmod1 is important for maintaining the length of the thin filament and the stability of the cardiomyocyte sarcomere structure. Tmod1 knockout disrupts the development of the heart chambers, with subsequent death of the embryo [13]. Using a culture of cardiomyocytes, it was shown that overexpression of Tmod1 leads to the formation of short thin filaments, while its absence disrupts myofibrillogenesis [14,15].

Experiments on Xenopus showed that Tmod4 is required in myofibrillogenesis in skeletal muscles [16]. In *Danio rerio* with a knockout of the *TMOD4* gene, a significant decrease in the number of myofibrils and impaired muscle force generation were observed [17]. Using a mouse model with *TMOD1* gene knockout, it was shown [18,19] that Tmod isoforms directly affect the formation of myosin cross-bridges and skeletal muscle force. Knockout of the *TMOD1* gene resulted in the replacement of Tmod1 by Tmod3/4 and a decrease in the mobility of the Tpm strand on the actin filament, reducing the number of cross-bridges in a strongly bound state and the force developed by the fiber [18,19]. It should be noted that in skeletal muscle, unlike the heart, *TMOD1* knockout does not affect the length of the thin filament, which means that the effect of Tmod on the regulation of actin–myosin interactions occurs through a change in the mobility of the Tpm strand [19].

The Tmod1 molecule (~40 kDa) can be divided into two parts [20,21,22]. The N-terminal domain of the Tmod1 molecule is predominantly unstructured. According to NMR data, only amino acid (a.a.) residues 24–35 form an α-helix. The N-terminal domain contains three functional sites: two tropomyosin (Tpm)-binding sites (a.a. residues 1–38, TMBS1, and 109–144, TMBS2) and one Tpm-dependent actin-capping site (a.a. residues 48–92, ABS1) [22,23,24,25,26,27,28,29]. The C-terminal part of the molecule is packed into a leucine-rich repeat (LRR) domain-containing second actin-binding site (ABS2) [30,31,32,33]. Using cultures of cardiomyocytes, it has been shown that the LRR domain is required for Tmod1 to bind to the actin filament’s minus end. Deletion of the LRR domain and changes in its structure due to point mutations lead to a complete loss of Tmod1 at the pointed end of the actin filament in the sarcomere of cardiomyocytes [21,34].

Due to the presence of two Tpm-binding sites, the Tmod1 molecule binds two Tpm dimers at the pointed end of the actin filament [27]. The N-terminal part of the Tpm molecule plays an important role in the interactions of Tmods with the minus end of the actin filament [22]. Tropomyosin enhances the binding of Tmod1 to the actin filament by three to four orders of magnitude [9,25,27,35,36].

The question of the influence of Tmod1 on the regulation of actin–myosin interactions in the myocardium remains open. The importance of Tmod for myocardial contraction was demonstrated by the discovery of a mutation that causes dilated and restrictive cardiomyopathy [37]. Here, we studied the effects of Tmod on the actin–myosin interaction in the myocardium using an in vitro motility assay. For the functioning of Tmod in striated muscles, the interaction of Tmod with Tpm is important. It was previously shown that the Tmod1 and Tmod3 isoforms bind Tpm isoforms with different affinities [18,31,38]. In the myocardium, Tpm1.1 (αTpm) and Tpm1.2 (κTpm) are expressed. Tpm 1.2 is the result of alternative splicing of the TPM1 gene [39]. In Tpm1.2 mRNA, exon 2b is replaced by exon 2a, which corresponds to ~ 40 amino acid residues (from a.a. 39 to a.a. 77) in the Tpm molecule [39]. Activation of Tpm1.2-containing thin filaments has been shown to depend on myosin [40].

It can be assumed that the binding of Tmod with the Tpm isoforms in the myocardium specifically modulates the actin–myosin interaction. To test this assumption, we studied the effects of Tmod on the interactions of cardiac myosin with actin using an in vitro motility assay. We assessed the interactions of Tmod with cardiac Tpm isoforms using cross-linking with glutaraldehyde, a pull-down assay, and size-exclusion chromatography. It can be assumed that the regulatory effect of Tmod on actin–myosin interactions depends on Tpm isoforms. We compared the effects of Tmod1 on the interactions of myosin with thin filaments containing Tpm1.1 and Tpm1.2. In addition, we tested whether the effect of Tmod on actin–myosin interactions in the myocardium depends on Tmod isoforms. For this, we used Tmod4, which is normally expressed in skeletal muscles.

## 2. Materials and Methods

### 2.1. Experimental Design, Animal Handling, and Ethics Requirements

A public corporation in Kamensk-Uralsky provided a sheep heart. Directive 2010/63/EU of the European Parliament was followed in the treatment of the rats used in the present study. The experimental protocol was approved by The Animal Care and Use Committee of the Institute of Immunology and Physiology. Unless otherwise indicated, Merck & Co. Inc. (Rahway, NJ, USA) was the supplier of all chemicals and reagents.

The institutional vivarium was occupied by 10-week-old male Wistar rats (250–300 g) that were provided free access to food (Delta Feeds LbK 120 S-19, BioPro, Novosibirsk, Russian) and water. An intramuscular injection of 2% Xylazine (1 mL/kg body weight, Alfasan, Woerden, The Netherlands) and Zoletil-100 (0.3 mL/kg body weight, Virbac, Carros, France) was used to anesthetize the rats. Then, heparin (5000 IU/kg, Ellara, Pokrov, Russian) was added, and the rats were euthanized through exsanguination. After removing the hearts, the left ventricles were frozen in portions for myosin isolation and stored at −86 °C.

### 2.2. Protein Extraction and Purification

Human Tpm1.1 and Tpm1.2 were expressed in *E. coli* C41(DE3) as previously described [40]. The Tpm1.1 and Tpm1.2 had Ala-Ser N-terminal extensions to mimic the naturally occurring acetylation of native Tpm [41]. A recombinant human cardiac Tn complex composed of TnI, TnT, and TnC was provided by HyTest (Turku, Finland) (Cat.# 8ITCR). Rat Tmod1 was obtained as described by Kostyukova and her co-authors [42,43]. CDSs of human Tmod1 and Tmod4 were obtained from Cloning Facility (Moscow, Russia) in an EV expression vector. All constructs had a 6X N-terminal His-tag. The Tmods were expressed in *E. coli* C41 (DE3) cells. A night culture was inoculated in 1 L of LB medium in 30 mM HEPES buffer (pH 7.3) with 100 mM NaCl and grew up to an optical density of 0.6 at 37 °C. Then, expression was induced by 0.2 mM IPTG and continued for 4 h at 30 °C. The Tmods were purified on a HisTrap HP column (GE Healthcare, Chicago, IL, USA) using a linear imidazole gradient from 15 to 500 mM. The samples were analyzed using SDS-PAGE electrophoresis. The best fractions were collected, combined, and loaded on a HiLoad 16/600 Superdex 200 pg column for additional purification. Finally, the Tmods were dialyzed against 30 mM HEPES-Na and 100 mM NaCl (pH 7.3) and stored at −80 °C.

The left ventricles and atria of the sheep and rat hearts were subjected to myosin extraction [44]. The atrial myosin from the sheep heart was composed of atrial light chain (LC) and 80% α-myosin heavy chain (MHC) with 20% β-MHC (Figure S1 in the Supplementary Materials). The sheep ventricular myosin entirely comprised β-MHC and ventricular light chain (VC). The rat ventricular myosin contained 90% α-MHC, 10% β-MHC, and ventricular LC. The rat atrial myosin contained 90% α-MHC, 10% β-MHC, and atrial LC. Native thin filaments (NTFs) were extracted from the sheep left ventricles [45]. Standard procedures [46] were utilized to prepare rabbit skeletal muscle actin, which was then polymerized and labeled with TRITC-phalloidin at a 2-fold molecular excess.

### 2.3. Chemical Cross-Linking Between Tmod and Tropomyosin

Tpm and Tmod samples were chemically cross-linked using an aqueous solution of glutaraldehyde (TED PELLA, Redding, CA, USA) in a 30 mM HEPES buffer, pH 7.3, with 200 mM NaCl. The concentrations of glutaraldehyde were 0.008% and 0.002%. The Tpm1.1 and Tpm1.2 concentrations were 0.2 mg/mL, and the Tmod1 and Tmod4 concentrations were 0.12 mg/mL. The cross-linking was performed for 15 and 60 min at 30 °C. After that, the samples were analyzed by SDS-PAGE in 12.5% gel.

### 2.4. Analytical Size-Exclusion Chromatography

Size-exclusion chromatography was performed using a Varian ProStar 3250 system (Varian, Belrose, Australia) on a Superose 6 Increase 10/300 GL column (GE Healthcare, Stockholm, Sweden) equilibrated by a 50 mM HEPES-Na buffer (pH 7.3) with 150 mM NaCl and 1 mM DTT. The sample volume was 100 µL with 30 µM Tpm and Tmod1 in all elution profiles with Tmod1 and 26 µM Tpm and 13 µM Tmod4 in all elution profiles with Tmod4. The elution profiles were recorded at 280 nm with a 0.5 mL/min flow rate for all experiments. The following protein standards were used: thyroglobulin (669 kDa), ferritin (440 kDa), aldolase (158 kDa), conalbumin (75 kDa), ovalbumin (43 kDa), and carboanhydrase (29 kDa), with RNAse (13.7 kDa) used for column calibration.

### 2.5. Pull-Down Assay

Experiments were performed by adding an affinity IMAC Ni-charged resin (Bio-Rad, Hercules, CA, USA) to Tpm/Tmod complexes. Before the experiments, the resin was washed three times by MQ water, then three times by a 30 mM HEPES-Na buffer, pH 7.3. Tmod1 and Tmod4 were incubated for 20 min in a 30 mM HEPES-Na buffer with 200 mM NaCl, pH 7.3, with increasing concentrations of Tpm1.1 and Tpm1.2 to the concentration ratio of 1:4 for Tmod/Tpm. After this, the resin was added to the complexes, and they were mixed and incubated for 5 min. The resin was pelleted by centrifugation and washed by a 30 mM HEPES-Na buffer with 200 mM NaCl, pH 7.3. Then, the resin was pelleted again and washed by 50 mM HEPES-Na, 300 mM NaCl, and 500 mM imidazole (pH 7.3). SDS-PAGE was used to analyze the supernatant probes. Protein bands were analyzed by ImageJ2 software (Scion, Frederick, MD, USA). For each Tpm/Tmod complex, three independent experiments were conducted.

### 2.6. In Vitro Motility Assay

The in vitro motility assay was carried out in accordance with a previous description [40,47,48]. Myosin (300 µg/mL) in an AB buffer (25 mM KCl, 25 mM imidazole, 4 mM MgCl_2_, 1 mM EGTA, and 20 mM DTT, pH 7.5) with 0.5 M KCl was loaded into a flow cell. After 2 min, 0.5 mg/mL BSA was added for 1 min. F-actin without labeling was added to an AB buffer with 2 mM ATP and left for 5 min. In order to produce regulated thin filaments, TRITC-phalloidin-labeled F-actin was added to the cell for 5 min. AB buffer was used to wash out the thin filaments that were not bound. The Maxchelator program (http://www.stanford.edu/~cpatton/webmaxc/webmaxcS.html (accessed on 1 November 2018)) was used to calculate the appropriate Ca^2+^ concentration. Finally, the cell was washed with an AB buffer containing 0.5 mg/mL BSA, an oxygen scavenger system, 20 mM DTT, 2 mM ATP, 0.5% methylcellulose, 100 nM Tpm/Tn, and Ca^2+^ ions. In each flow cell, ten 30 s image sequences were recorded at 30 °C from different fields containing ~30–50 thin filaments. GMimPro2023 software [49] was used to measure the sliding velocities of the filaments. We measured the sliding velocities of filaments that were at least 2 μm long and moved for at least 10 frames. We found that adding Tmod did not affect the lengths of the F-actin and thin filaments (Figure S2 and Table S1 in the Supplementary Materials).

To study the effect of Tmod on the Ca^2+^ regulation of the actin–myosin interaction, we analyzed the dependence of the sliding velocity of the thin filaments over atrial and ventricular myosin on the Ca^2+^ concentration. Tmod was added to an AB buffer containing ATP. The means of individual experiments were fitted using the Hill equation: V = V_max_ × (1 + 10^*h*(pCa- pCa50)^)^−1^, where V and V_max_ are the velocity and the maximal velocity at the saturating Ca^2+^ concentration, respectively; pCa_50_ (i.e., the Ca^2+^ sensitivity) is the pCa at which the velocity is half-maximal; and *h* is the Hill cooperativity coefficient. The parameters of individual experiments were averaged.

To assess the impact of Tmod on cross-bridge cooperation, the sliding velocities of thin filaments were evaluated in relation to the concentration of myosin added to the flow cell. This dependence was fitted with the modified Hill equation [50] V = V_max_ × *c*^h^ × (*c*_50_^h^ + *c*^h^)^−1^, where V_max_ is the maximal sliding velocity, *C* is the myosin concentration, *C*_50_ is the concentration required to achieve the half-maximal velocity, and h is the Hill coefficient.

Data analysis was performed using Excel 16 (Microsoft Corp., Redmond, WA, USA) and Origin 8.0 (Origin Lab, Northampton, MA, USA). All values were expressed as means ± SDs after three repetitions of the experiments. The Mann–Whitney U test was used to estimate the statistical significance of the characteristics of the Hill equation. In experiments where the effect of Tmod on the sliding velocity was studied, we measured the velocities of about 100 filaments, and comparisons of the filament sliding velocities at different Tmod concentrations were performed using Student’s *t*-test.

## 3. Results

### 3.1. The Effect of Tmod on the Sliding Velocity of F-Actin–Tpm Filaments on Sheep Myosin in the in Vitro Motility Assay

**Figure 1:**
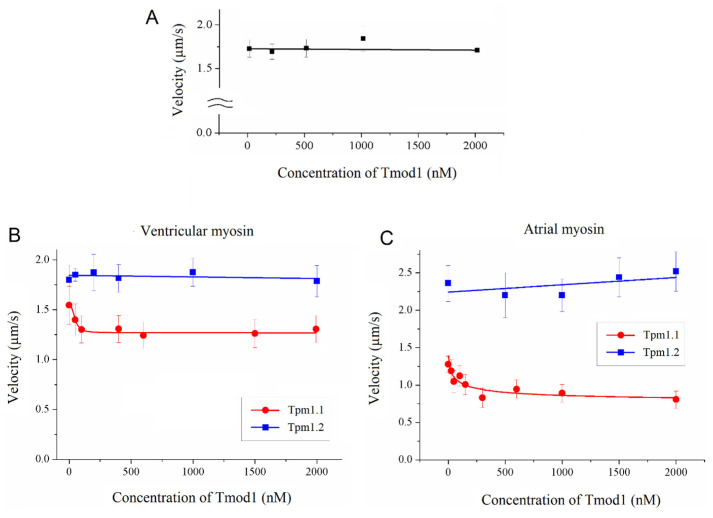
The effect of Tmod1 on the sliding velocities of F-actin and F-actin–Tpm filaments on sheep cardiac myosin in the in vitro motility assay. (**A**) The dependence of the sliding velocity of F-actin on the concentration of Tmod1 over sheep ventricular myosin. (**B**,**C**) The dependence of the sliding velocities of F-actin–Tpm filaments containing Tpm1.1 and Tpm1.2 on the Tmod1 concentration on sheep ventricular (**B**) and atrial (**C**) myosin. The experimental data for Tmod1 with F-actin–Tpm1.1 filaments and F-actin–Tpm1.2 filaments are approximated by exponential and linear functions, respectively. The experimental values are presented as means ± SDs.

First, we measured the sliding velocity of F-actin over sheep ventricular myosin at Tmod1 and Tmod4 concentrations from 50 nM to 2000 nM. Tmod1 (Figure 1A) and Tmod4 did not affect the F-actin velocity. To study the effect of Tmod on the interaction of F-actin with Tpm, we analyzed the dependence of the sliding velocity of F-actin–Tpm filaments on the concentration of Tmod1. Tmod1 had different effects on the velocities of F-actin–Tpm filaments containing Tpm1.1 and Tpm1.2. Tmod1 slightly dose-dependently decreased the sliding velocity of F-actin–Tpm1.1 filaments on sheep ventricular and atrial myosins and did not affect the sliding velocity of F-actin–Tpm1.2 filaments (Figure 1B,C).

**Figure 2:**
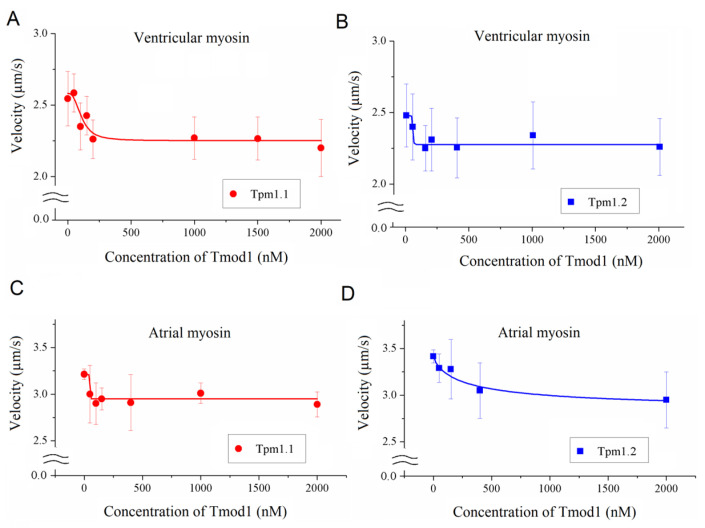
The effect of Tmod1 on the maximum sliding velocities at pCa4 of thin filaments containing F-actin, Tpm, and Tn on sheep cardiac myosin in the in vitro motility assay. (**A**–**D**) The dependence of the maximum sliding velocities (pCa4) of thin filaments containing Tpm1.1 (**A**,**C**) and Tpm1.2 (**B**,**D**) on sheep ventricular (**A**,**B**) and atrial (**C**,**D**) myosin on the Tmod1 concentration. The experimental data in (**A**–**D**) are approximated by exponential functions. The experimental values are presented as means ± SDs.

At the saturating calcium concentration (pCa4) with ventricular myosin, Tmod1 slightly decreased the maximum sliding velocities of thin filaments reconstructed from F-actin, troponin, and Tpm1.1 (Figure 2A) and Tpm1.2 (Figure 2B). With atrial myosin, Tmod1 decreased the maximum sliding velocities of thin filaments containing Tpm1.1 and Tpm1.2 (Figure 2C,D).

### 3.2. The Effect of Tmod on the Calcium Regulation of the Actin–Myosin Interaction

**Figure 3:**
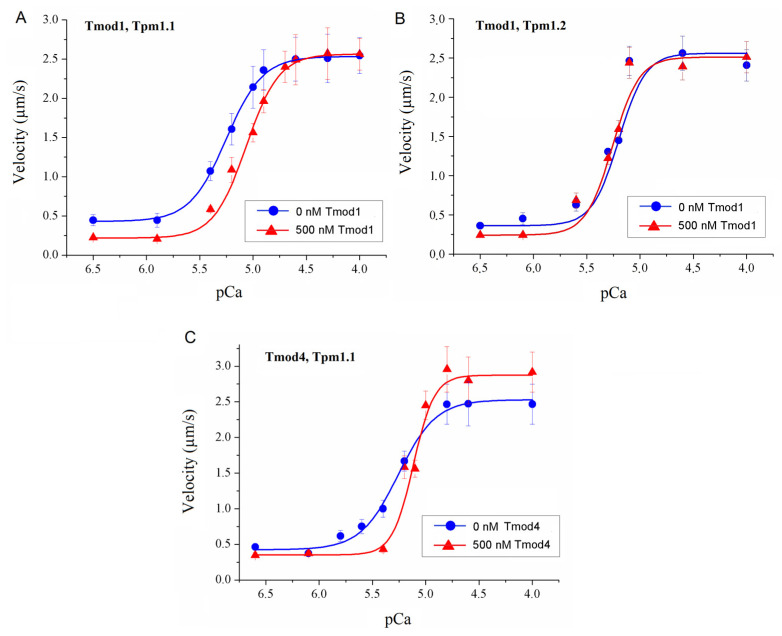
The effect of Tmod1 on the maximum sliding velocities at pCa4 of thin filaments reconstructed from F-actin, troponin, and Tpm on sheep cardiac myosin in the in vitro motility assay. (**A**,**B**) The effect of 500 nM Tmod1 on the calcium dependence of the sliding velocities of thin filaments containing Tpm1.1 (**A**) and Tpm1.2 (**B**) over sheep ventricular myosin. (**C**) The effect of 500 nM Tmod4 on the calcium dependence of the sliding velocity of thin filaments containing Tpm1.1 over sheep ventricular myosin. The experimental values are means ± SDs. The calcium dependence of the filament sliding velocity was approximated by the Hill equation; the values of the parameters are given in Table 1.

Ventricular and atrial myosin differ in their functional characteristics [47,48,51,52]. Therefore, in the in vitro motility assay we used ventricular and atrial myosin. We studied the effect of 500 nM Tmod on the Ca^2+^ regulation of the actin–myosin interaction by analyzing the Ca^2+^ dependence of the sliding velocities of regulated thin filaments reconstructed from F-actin, Tpm, and troponin on sheep ventricular and atrial myosin in the in vitro motility assay. With ventricular myosin, Tmod1 reduced the Ca^2+^ sensitivity of the velocity of thin filaments containing Tpm1.1 (Figure 3A and Table 1) and did not affect the Ca^2+^ dependence of the velocity of thin filaments with Tpm1.2 (Figure 3B and Table 1). Tmod4 decreased the Ca^2+^ sensitivity of the velocity of thin filaments containing Tpm1.1 but increased both the Hill cooperativity coefficient and the maximum filament velocity on ventricular myosin (Figure 3C and Table 1).

**Figure 4:**
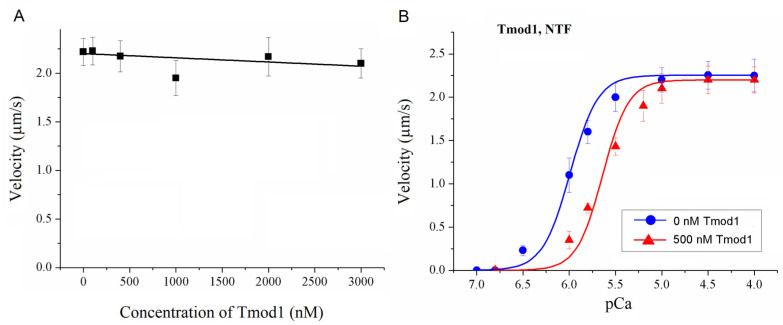
The effect of Tmod1 on the interaction of sheep ventricular myosin with native thin filaments. (**A**) The dependence of the maximum sliding velocity (pCa4) of native thin filaments (NTFs) over sheep ventricular myosin on the Tmod1 concentration in the in vitro motility assay. NTFs and myosin were extracted from the sheep left ventricle. The experimental data are presented as means ± SDs and approximated by a linear function. (**B**) The effect of 500 nM Tmod1 on the calcium dependence of the sliding velocity of NTFs on sheep ventricular myosin in the in vitro motility assay. The experimental values are means ± SDs. The calcium dependence of the filament sliding velocity was approximated by the Hill equation; the values of the parameters are given in Table 1.

Next, we analyzed the effect of Tmod1 on the Ca^2+^ regulation of the actin–myosin interaction using myosin and native thin filaments (NTFs) extracted from the left ventricle of a sheep. Tmod1 did not affect the maximal NTF sliding velocity on myosin (Figure 4A) and significantly decreased the Ca^2+^ sensitivity of the NTF velocity (Figure 4B and Table 1).

Note that the main effect, both in the case of the reconstructed thin filament with recombinant Tpm and in the case of NTFs with natural acetylation of Tpm, is a decrease in the calcium sensitivity of the actin–myosin interaction. According to previous studies [25], the Ala-Ser extension does not significantly affect the interaction of Tpm with Tmod. Our results obtained with NTFs indirectly indicate that the Ala-Ser extension does not affect Tmod’s effects on the actin–myosin interaction.

**Figure 5:**
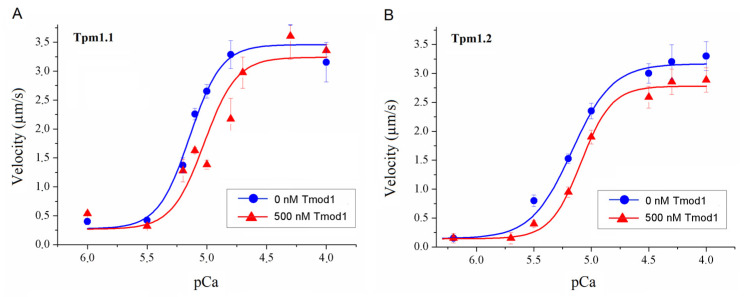
The effect of Tmod1 on the calcium dependence of the sliding velocity of thin filaments on sheep atrial myosin in the in vitro motility assay. (**A**) The effect of 500 nM Tmod1 on the calcium dependence of the sliding velocity of thin filaments containing Tpm1.1 on sheep atrial myosin. (**B**) The effect of 500 nM Tmod1 on the calcium dependence of the sliding velocity of thin filaments containing Tpm1.2 on sheep atrial myosin. The experimental values are means ± SDs. The calcium dependence of the filament sliding velocity was approximated by the Hill equation, and the values of the parameters are given in Table 2.

With sheep atrial myosin, Tmod1 reduced the Ca^2+^ sensitivity of the velocities of thin filaments with both Tpm1.1 and Tpm1.2 (Figure 5 and Table 2).

**Figure 6:**
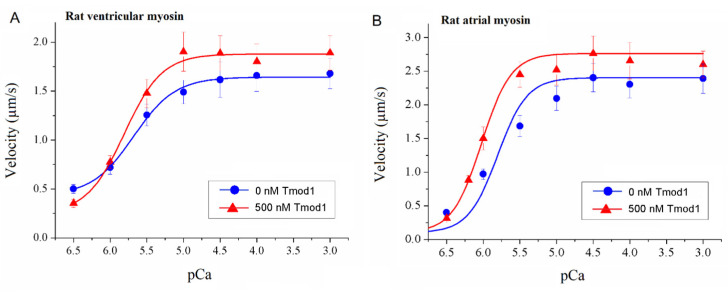
The effect of rat Tmod1 on the calcium dependence of the sliding velocity of thin filaments on rat cardiac myosin in the in vitro motility assay. (**A**) The effect of 500 nM rat Tmod1 on the calcium dependence of the sliding velocity of thin filaments containing Tpm1.1 on rat ventricular myosin. (**B**) The effect of 500 nM rat Tmod1 on the calcium dependence of the sliding velocity of thin filaments containing Tpm1.1 on rat atrial myosin. The experimental values are means ± SDs. The calcium dependence of the filament sliding velocity was approximated by the Hill equation, and the values of the parameters are given in Table 3.

Myosins of large and small animals differ in their mechanical and kinetic properties [53,54] and in their effects on the activation of the thin filament [40]. Therefore, we compared the effects of Tmod1 on the calcium regulation of the actin–myosin interaction with sheep and rat myosin. In an experiment with rat myosin, we used rat Tmod1. In contrast to sheep myosin and human Tmod1, rat Tmod1 increased both the maximal thin filament sliding velocities on rat atrial and ventricular myosin and the calcium sensitivity of the actin–myosin interaction (Figure 6 and Table 3).

### 3.3. Influence of Tmod on Cross-Bridge–Cross-Bridge Cooperativity of Myosin Interaction with Filaments

**Figure 7:**
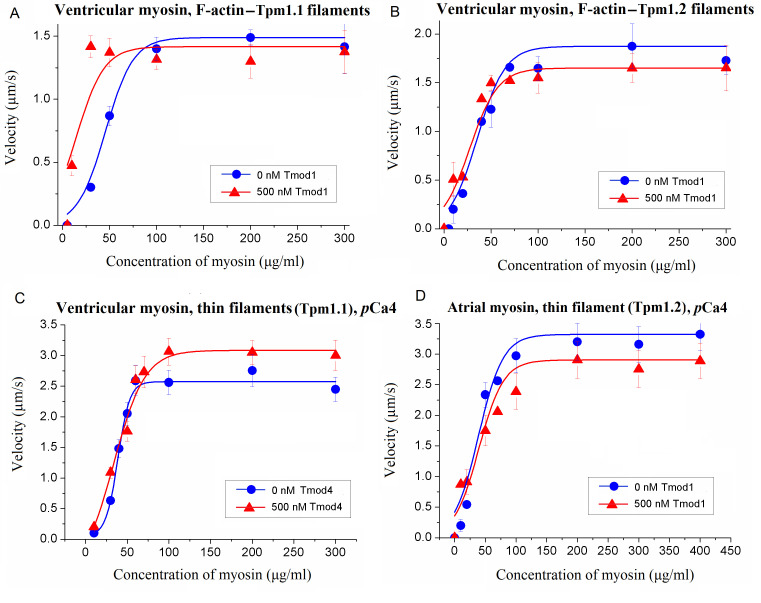
The effect of Tmod1 on cross-bridge–cross-bridge (Xb-Xb) cooperativity. (**A**,**B**) The effect of 500 nM Tmod1 on the Xb-Xb cooperativity of the interactions of sheep ventricular myosin with F-actin–Tpm filaments containing Tpm1.1 (**A**) and Tpm1.2 (**B**). (**C**) The effect of 500 nM Tmod4 on the Xb-Xb cooperativity of the interaction of sheep ventricular myosin with F-actin–Tpm filaments containing Tpm1.1. (**D**) The effect of 500 nM Tmod1 on the Xb-Xb cooperativity of the interaction of sheep atrial myosin with F-actin–Tpm filaments containing Tpm1.1. The experimental values are presented as means ± SDs. The experimental values are approximated by the Hill equation.

Cooperative mechanisms play an important role in cardiac muscle contraction, one of which is cross-bridge–cross-bridge (Xb-Xb) cooperativity. To assess the effect of Tmod on Xb-Xb cooperativity, we analyzed the dependence of the F-actin–Tpm filament velocity on the concentration of sheep ventricular myosin loaded into the flow cell. Tmod1 enhanced the Xb-Xb cooperativity of the interaction of myosin with F-actin–Tpm1.1 filaments. Tmod1 decreased the ventricular myosin concentration required to achieve the half-maximal filament velocity (C_myosin_) (Figure 7A and Table 4) but did not affect it with F-actin–Tpm1.2 filaments (Figure 7B and Table 4). At the saturating calcium concentration (pCa 4), Tmod1 and Tmod4 did not affect the Xb-Xb cooperativity of the interactions of thin filaments with ventricular and atrial myosin (Figure 7C,D and Table 4).

**Figure 8:**
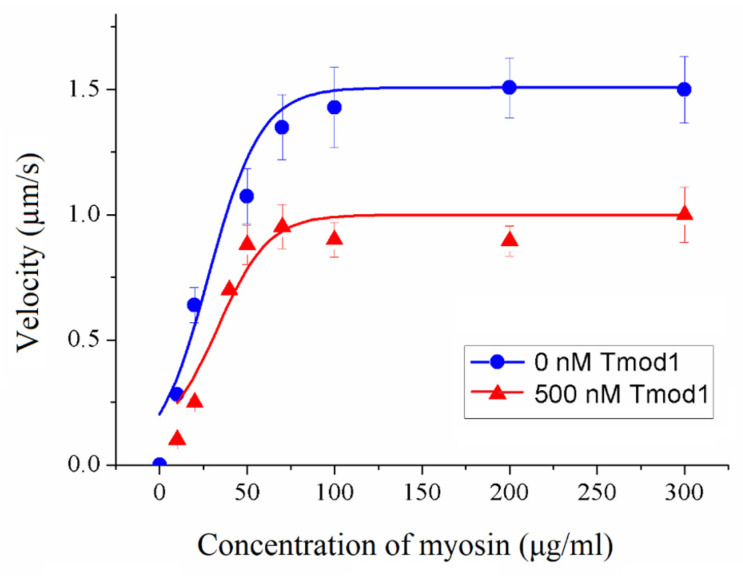
The effect of 500 nM Tmod1 on the cross-bridge–cross-bridge cooperativity of the interaction of sheep ventricular myosin with thin filaments at a non-saturating calcium concentration (pCa5.2). The experimental values are presented as means ± SDs and approximated by the Hill equation.

Using sheep ventricular myosin, we studied the effect of Tmod1 on the Xb-Xb cooperativity of the actin–myosin interaction at a non-saturating calcium concentration (pCa 5.2). We found that Tmod1 did not affect the myosin concentration at which the sliding velocity of the thin filaments was half-maximal, but it decreased the sliding velocity of the filaments by 30% (Figure 8 and Table 4).

### 3.4. Chemical Cross-Linking

**Figure 9:**
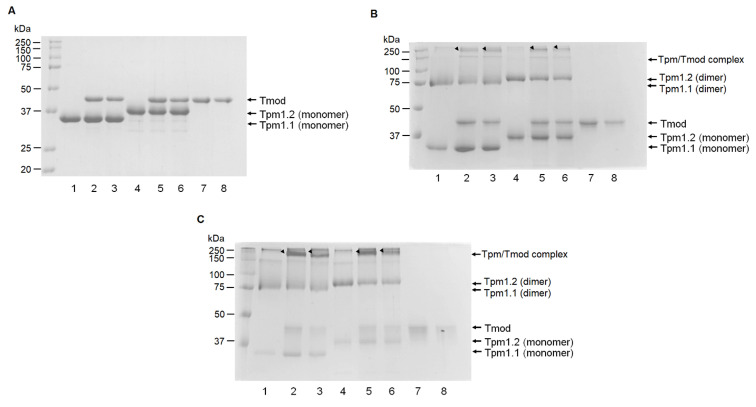
The chemical cross-linking of Tpm and Tmod isoforms. (**A**) Samples subjected to cross-linking before adding glutaraldehyde. (**B**) Chemical cross-linking by 0.002% glutaraldehyde for 15 min. (**C**) Chemical cross-linking by 0.008% glutaraldehyde for 15 min. Lanes: 1—Tpm1.1; 2—Tpm1.1 with Tmod1; 3—Tpm1.1 with Tmod4; 4—Tpm1.2; 5—Tpm1.2 with Tmod1; 6—Tpm1.2 with Tmod4; 7—Tmod1; 8—Tmod4. Black arrows mark the positions of Tpm/Tmod complexes. Original images can be found in Supplementary Materials.

To study the formation of complexes between Tpm and Tmod isoforms, we applied chemical cross-linking with glutaraldehyde. We found that both Tpm isoforms are able to form complexes with Tmod1 and Tmod4 (Figure 9). The apparent molecular weight of such complexes is around 160–200 kDa.

### 3.5. Analytical Size-Exclusion Chromatography

**Figure 10:**
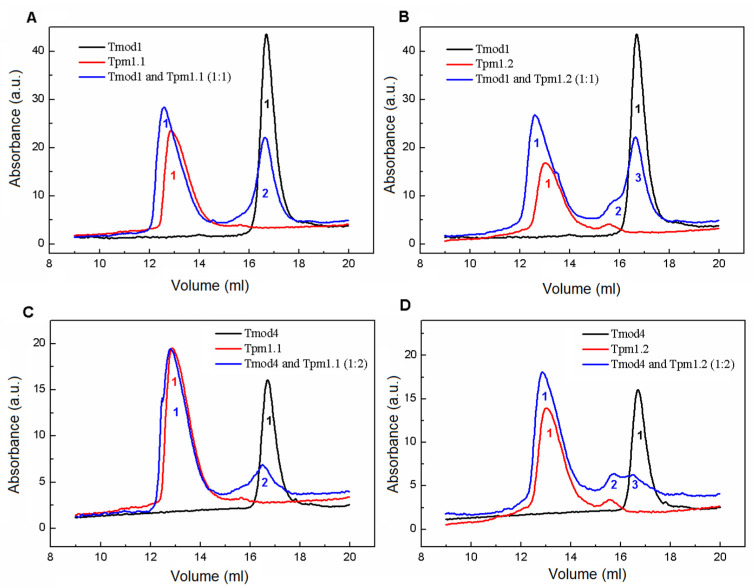
Analytical size-exclusion chromatography of Tmod1, Tmod4, Tpm1.1, Tpm 1.2, and their mixtures loaded on a Superose 6 Increase 10/300 GL column. (**A**) The elution profiles of Tmod1, Tpm1.1, and a mixture of Tmod1 and Tpm1.1 at a concentration ratio of 1:1. (**B**) The elution profiles of Tmod1, Tpm1.2, and a mixture of Tmod1 and Tpm1.2 at a concentration ratio of 1:1. (**C**) The elution profiles of Tmod4, Tpm1.1, and a mixture of Tmod4 and Tpm1.1 at concentration ratio of 1:2. (**D**) The elution profiles of Tmod4, Tpm1.2, and a mixture of Tmod4 and Tpm1.2 at a concentration ratio of 1:2. The digits indicate peak numbers; the peak parameters are presented in Table 5 and Table 6.

To study the interactions of the Tmod and Tpm isoforms, we used analytical size-exclusion chromatography (SEC). First, SEC profiles were obtained for individual proteins of Tmod1, Tmod4, Tpm1.1, and Tpm1.2. The Tmod1 profiles were presented with one symmetrical peak with an elution volume of 16.7 mL, which corresponded to a molecular mass of approximately 40 kDa (Figure 10A,B and Table 5). This fact allows us to conclude that Tmod1 is a monomer. The same data were obtained for Tmod4 (Figure 10C,D and Table 6). More complicated data were obtained for Tpm1.1 (Figure 10A,C) and Tpm1.2 (Figure 10B,D). Elution profiles were presented with single asymmetrical peaks with elution volumes of 12.9 mL for Tpm1.1 and 13.0 mL for Tpm1.2, which corresponded to approximately 600–700 kDa. Two factors could cause such deviations in elution profiles. First, although the SEC experiments were performed in a buffer containing 150 mM NaCl, a Tpm end-to-end interaction leads to cord formation under these experimental conditions. The other factor could be the shape of Tpm, which was far from a globular protein. We are inclined to favor the second option because it is hard to imagine that the formation of a Tpm strand could lead to a population of rods with similar molecular masses.

The elution profile of an equimolar mixture of Tpm1.1 and Tmod1 (Figure 10A) was presented with two major peaks with elution volumes of 12.6 and 16.7 mL (Table 5). The first peak corresponded to the complex of Tpm1.1 and Tmod1, and the second peak corresponded to residual amounts of Tmod1. A two-fold decrease in the second peak indicated that under these experimental conditions, Tmod1 interacted with Tpm1.1 at a ratio close to 1:2. The results obtained for an equimolar mixture of Tpm1.2 and Tmod1 (Figure 10B) were very similar to those for Tpm1.1 and Tmod1. However, a small shoulder appeared in the elution profile near to Tmod1 peak (Figure 10B, peak 2). We suppose that this shoulder corresponded to a complex of Tmod1 with a small amount of Tpm1.2 proteolysis fragments. Tpm1.2 still contained proteolytic fragments that could not be completely removed. They can be seen in Figure 9, lanes 4–6. Other preparations had higher purity, but minor changes in the elution profiles may have been caused by trace amounts of protein impurities. Overall, no differences in the interactions of Tmod1 with Tpm1.1 and Tpm1.2 were found under these experimental conditions.

The elution profile of a mixture of Tmod4 and Tpm1.1 with a concentration ratio of 1:2 was presented with two peaks (Figure 10C) with elution volumes of 12.8 and 16.5 mL (Table 6). These peaks were very close at their maxima to the peaks of free Tpm1.1 and Tmod4. However, a decrease in the absorption value of the Tmod4 peak indicated the formation of a complex between Tmod4 and Tpm1.1. It is noteworthy that the formation of the Tmod4/Tpm1.1 complex did not lead to a shift in the first peak of the elution curve (Figure 10C). The difference in the hydrodynamic radii of the Tpm1.1/Tmod4 and Tpm1.1/Tmod1 complexes may indicate a distinction in the interactions of the Tpm and Tmod isoforms. The elution profile obtained for the Tpm1.2/Tmod4 complex was similar to that of the Tpm1.1/Tmod4 complex (Figure 10D). As for the previous profile obtained for the mixture of Tmod1 and the Tpm1.2 isoform, we assume that the appearance of peak 2 (Figure 10D) corresponds to a complex of Tmod4 with small amounts of Tpm1.2 proteolysis fragments.

Thus, the formation of complexes of both studied Tmod isoforms with cardiac isoforms Tpm1.1 and Tpm1.2 indicates firm interactions between these proteins even without fibrillar actin.

### 3.6. Formation of Tmod/Tpm Complexes Determined by Pull-Down Assay

**Figure 11:**
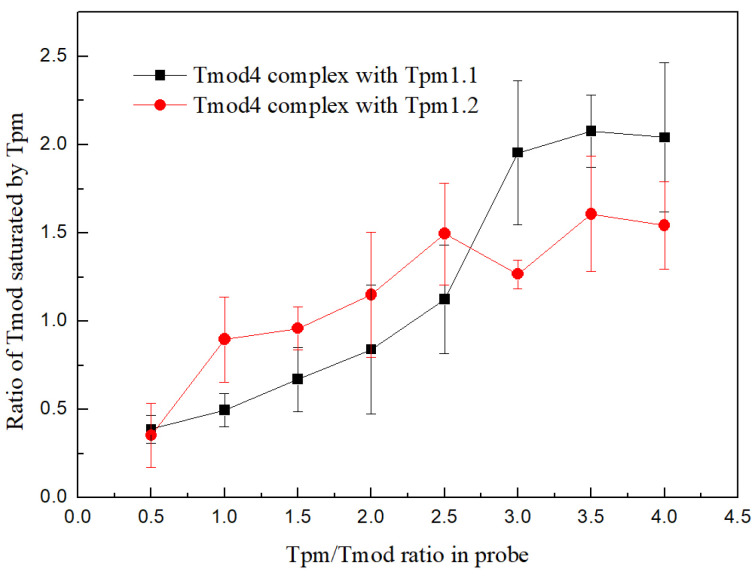
The interactions of Tmod4 with Tpm1.1 and Tpm1.2 measured by the pull-down assay. This graph shows the dependence of Tmod4 saturation on Tpm1.1 (black line) and Tpm1.2 (red line) added to the sample in different concentrations. The Tmod4 concentration was 6 µM in all samples, and the Tpm concentrations ranged from 3 to 24 µM. The experimental values are presented as means ± SDs.

To study complex formation between Tmod and different cardiac Tpm isoforms, we used a pull-down assay. We found that the saturation patterns of the Tmod4 complexes with tropomyosin were similar for the two tropomyosin isoforms Tpm1.1 and Tpm1.2 (Figure 11). All curves had growth and a sigmoidal shape, and they reached saturation at Tpm/Tmod ratios of 2.5 for Tpm1.2 and 3 for Tpm1.1 (Figure 11). The deviations and accuracy of this method made these values very close, and they did not differ from each other. Unfortunately, saturation curves for Tmod1 could not be obtained because tropomyosin washed it off the resin.

## 4. Discussion

Previously, it was found that in skeletal muscle, Tmod not only regulates the length and stability of a thin filament but also affects the movement of the Tpm strand along the actin filament and the formation of myosin cross-bridges [18,19]. The replacement of Tmod1 by Tmod3/4 led to decreases in the mobility of Tpm on the actin filament and in the number of myosin cross-bridges in a strongly bound state. In the heart, a change in the expression of Tmod1 and Lmod2 leads to changes in the length of the thin filament and the structure of the sarcomere [4,5,6], which makes it difficult to study the participation of Tmod1 in the activation of the thin filament in transgenic models. Therefore, to study the functional effects of Tmod on the actin–myosin interaction, we used the in vitro motility assay. In addition, we analyzed the formation of complexes between Tpm and Tmod isoforms with chemical cross-linking by glutaraldehyde, a pull-down assay, and analytical size-exclusion chromatography (SEC). We found that Tmod1 did not affect the sliding velocity of bare F-actin over myosin in the in vitro motility assay. However, the effects of Tmod on actin–myosin interactions were pronounced in the presence of Tpm or the Tpm/Tn complex on the actin filament. In other words, through the interaction of Tmod with Tpm, Tmod influences the interaction of myosin with the actin filament. Tmod’s effects on the actin–myosin interaction depend on the composition of the protein isoforms.

### 4.1. Isoform-Dependent Effects of Tmod on Actin–Myosin Interaction

We found that the effect of Tmod1 on the sliding velocity of F-actin–Tpm filaments depends on the Tpm isoforms. Tmod1 dose-dependently decreased the sliding of F-actin–Tpm1.1 filaments and had no effect on the velocity of F-actin–Tpm1.2 filaments (Figure 1). The effect of Tmod1 on the calcium regulation of the actin–myosin interaction also depended on the Tpm and myosin isoforms (Figure 3A,B and Figure 4). Tmod1 reduced the calcium sensitivity of thin filaments containing Tpm1.1 with ventricular and atrial myosin. The Tpm1.1 and 1.2 isoforms differ at 27 amino acid residues located between the 39th and 77th residues in the N-terminal part (Figure S3 in the Supplementary Materials) [39]. However, using the example of Tmod2, it was shown that Tmod interacts with 14 amino acid residues located in the N-end region of the Tpm molecule [22]. The difference in Tmod1’s effect on actin–myosin interactions with Tpm1.1 and Tpm1.2 can be explained by a long-range effect caused by the difference in the amino acid sequences of the N-terminal parts of these Tpm isoforms. A similar effect was previously described for myopathic Tpm mutations in the N-terminal part of this molecule [55].

In contrast to the NTF (Figure 4A), the reconstructed thin filament showed a tendency to decrease its sliding velocity with the addition of Tmod1 (Figure 2A). This difference may be explained by post-translational modifications of the NTF proteins, in particular, phosphorylation of Tpm, troponin T, and troponin I.

In addition to the Tmod and Tpm isoforms, the myosin isoform composition should also be considered. Ventricular and atrial myosins differ in the isoform compositions of heavy and light chains and their functional characteristics [47,48,51,52]. This myosin isoform composition affects the cooperativity regulation of the actin–myosin interaction. Fast-cycling atrial myosin activates the thin filament less efficiently than ventricular myosin, thus affecting the calcium regulation of the actin–myosin interaction [47,48]. Here, we found that the effect of Tmod1 on the actin–myosin interaction also depends on myosin isoforms. Tmod1 did not affect the calcium regulation characteristics of ventricular myosin with the thin filament containing Tpm1.2 (Figure 3B and Table 1) but decreased calcium sensitivity with atrial myosin (Figure 4B and Table 2). We previously discovered that atrial myosin activates thin filaments containing Tpm1.2 stronger than ventricular myosin [48]. On the one hand, myosin interacts with tropomyosin [56,57]. The differences in the S1 amino acid sequences of atrial and ventricular myosin can affect this interaction. On the other hand, the atrial and ventricular light chain isoforms also influence the actin–myosin interaction [47,48,52].

Tpm1.2 expression has been shown to be higher in the atria than in the ventricles [58]. We found that Tmod did not affect interactions of ventricular myosin with thin filaments containing Tpm1.2 and influenced interactions of atrial myosin with thin filaments containing Tpm1.2. Therefore, it can be assumed that the interaction of Tmod with Tpm1.2 may contribute to atrial contraction. Further study is needed to confirm this.

It has previously been shown that the Tmod1, Tmod3, and Tmod4 isoforms bind non-muscle Tpm isoforms and striated muscle Tpms with different affinities [18,31,38,59]. In addition, it was found that site 1 and site 2 bind Tpm with different affinities [36], and it can be assumed that this difference may influence the interactions of myosin with the thin filament. Using Tmod4, we tested whether differences in the amino acid sequences of the Tpm-binding sites of Tmod isoforms influence the calcium regulation of the actin–myosin interaction. The homologies of Tpm-binding sites 1 and 2 for Tmod1 and Tmod4 are ~60% and ~80%, respectively (Figure S4 in the Supplementary Materials). Unlike Tmod1, Tmod4 increased the maximum velocity of thin filaments containing Tpm1.1. All these results confirm that the features of the interaction of Tmod with Tpm depend on the isoforms of these proteins. Using SEC and a pull-down assay, we found that Tmod1 and Tmod4 interact in a similar manner with both Tpm1.1 and Tpm1.2 (Table 5 and Table 6 and Figure 11). However, we did not find any significant difference in the complex formation between Tmod and Tpm isoforms. Therefore, we can suppose that the differences in the results can be explained by the peculiarities of the interactions of filaments containing Tpm isoforms with myosin isoforms.

Myosins of large and small animals differ in their functional properties [53,60] and in their effects on the activation of the thin filament [40]. For example, it was shown that the calcium sensitivity of the interaction of mouse ventricular myosin with thin filaments containing Tpm1.2 is lower than that with thin filaments containing Tpm1.1 [39], whereas the calcium sensitivity of the interactions of porcine ventricular myosin with thin filaments containing Tpm1.2 and Tpm1.1 do not differ [40]. Using rat cardiac myosin and rat Tmod1 as an example, we showed that the effect of Tmod on the actin–myosin interaction may depend on the species-specific features of myosin. Unlike human Tmod1 with sheep myosin, rat Tmod1 increased both the maximal sliding velocities of thin filaments over rat ventricular and atrial myosin and the calcium sensitivity of the filament velocity (Figure 5). Different effects of interactions between sarcomere proteins in large and small animals were previously shown for Tpm1.2 [40]. The peculiarities of the functioning of sarcomeric proteins are a mechanism of adaptation to a certain contractile function of the heart of large and small animals..

### 4.2. The Mechanism of Tmod’s Influence on the Actin–Myosin Interaction

The above results allow us to conclude that Tmod has a direct effect on the actin–myosin interaction in the myocardium and is one of its regulatory components. What is the mechanism of Tmod’s influence on the actin–myosin interaction? Data from previous studies and our work suggest that Tmod affects the mobility of the Tpm cable along the surface of the actin filament. The mobility of the Tpm molecule on the actin filament is necessary for activation of the thin filament. During muscle relaxation, Tpm blocks myosin-binding sites on actin (the blocked state). When calcium enters the cytosol and is bound by troponin C, Tpm begins to move azimuthally along the actin surface, opening myosin-binding sites (the closed state). Attachment of myosin heads promotes propagation of Tpm movement to adjacent actin monomers and complete activation of the thin filament (the open or M state) [61]. Using mouse knockout models and experiments with X-ray diffraction, Ochala et al. [19] demonstrated that the interaction of Tmod with Tpm at the pointed end of the actin filament may affect Tpm movement and that this effect differs between the relaxed and activated states of the thin filament and spreads along its entire length due to a long-range effect. Namely, it affects the movement of the Tpm strand at the beginning of thin filament activation. The presence of such effects during the interaction of Tpm with Tmod was discovered in the work of Moraczewska et al. [55] using the example of myopathic mutation in middle part of the Tpm molecule.

Ochala et al. [19] assumed that to a greater extent, Tmod affects the movement of the Tpm strand along the surface of the actin filament in the initial stages of activation. This assumption was indirectly confirmed by the results of our experiments on the dependence of filament velocity on the myosin concentration. The formation of myosin cross-bridges is important for the activation of the thin filament [61,62,63,64]. We found that Tmod1 enhanced the Xb-Xb cooperativity of the interaction of myosin with F-actin–Tpm filaments (Figure 7A) in the absence of calcium but did not affect it at the saturating calcium concentration (Figure 7C). At a non-saturating calcium concentration, Tmod1 decreased the thin filament velocity (Figure 8), which indicated the number of cross bridges because the filament velocity was determined by the cross-bridge number in the in vitro motility assay. With an increase in the calcium concentration, the influence of Tmod was not significant compared to other processes of activation of the thin filament. Further research is required to explain the molecular mechanisms of Tmod’s participation in the activation of the thin filament.

## 5. Conclusions

Previously, using X-ray diffraction of single skinned skeletal muscle fibers, it was shown that Tmod affects the movement of the Tpm strand along the actin filament and the formation of myosin cross-bridges [35,36]. Here, with an in vitro motility assay and isolated sarcomeric proteins, we demonstrated that Tmod takes part in the regulation of the actin–myosin interaction in the myocardium and that Tmod’s effect on thin filament activation is determined by the Tpm and myosin isoforms. The specific interaction of the Tmod isoform with actin and tropomyosin may be important for the regulation of the actin–myosin interaction in cardiac and skeletal muscles.

## Figures and Tables

**Table 1:** The parameters of the Ca^2+^ dependence of the sliding velocities of thin filaments over sheep ventricular myosin in the in vitro motility assay.

Tmod Isoform	Filament	Tmod Concentration (nM)	V_max_ (µm/s)	*h*	pCa_50_
Tmod1	Tpm1.1	0	2.6 ± 0.1	1.2 ± 0.1	5.25 ± 0.01
		500	2.6 ± 0.1	1.5 ± 0.2	5.06 ± 0.02 *
Tmod4		0	2.5 ± 0.1	1.2 ± 0.1	5.26 ± 0.01
		500	2.9 ± 0.1 *	2.2 ± 0.2 *	5.12 ± 0.01 *
Tmod1	Tpm1.2	0	2.6 ± 0.1	1.7 ± 0.1	5.20 ± 0.01
		500	2.5 ± 0.1	1.8 ± 0.1	5.27 ± 0.02
Tmod1	NTF	0	2.3 ± 0.1	1.7 ± 0.1	5.99 ± 0.01
		500	2.2 ± 0.1	1.8 ± 0.2	5.64 ± 0.01 *

V_max_ is the maximum sliding velocity of thin filaments at a saturating Ca^2+^ concentration; *h* is the Hill cooperativity coefficient; and pCa_50_ is the Ca^2+^ concentration at which the velocity is half-maximal and the Ca^2+^ sensitivity. Simbol * indicates differences between the values of the parameters of the Hill equation with and without Tmod (Mann–Whitney U test, *p* < 0.05).

**Table 2:** The parameters of the Ca^2+^ dependence of the sliding velocities of thin filaments on sheep atrial myosin in the in vitro motility assay.

Tpm Isoform	Tmod1 Concentration (nM)	V_max_ (µm/s)	*h*	pCa_50_
Tpm1.1	0	3.4 ± 0.3	1.7 ± 0.2	5.14 ± 0.03
	500	3.2 ± 0.2	1.7 ± 0.1	5.02 ± 0.02 *
Tpm1.2	0	3.2 ± 0.2	1.4 ± 0.2	5.17 ± 0.01
	500	2.8 ± 0.1	1.8 ± 0.2	5.09 ± 0.03 *

V_max_ is the maximum sliding velocity of thin filaments at a saturating Ca^2+^ concentration; *h* is the Hill cooperativity coefficient; and pCa_50_ is the Ca^2+^ concentration at which the velocity is half-maximal and the Ca^2+^ sensitivity. Simbol * indicates differences between the values of the parameters of the Hill equation with and without Tmod (Mann–Whitney U test, *p* < 0.05).

**Table 3:** The parameters of the Ca^2+^ dependence of the sliding velocities of thin filaments over rat cardiac myosin in the in vitro motility assay.

Myosin	Rat Tmod1 Concentration (nM)	V_max_ (µm/s)	*h*	pCa_50_
LV	0	1.64 ± 0.03	1.0 ± 0.1	5.68 ± 0.04
	500	1.87 ± 0.02 *	1.2 ± 0.2	5.81 ± 0.01 *
LA	0	2.40 ± 0.01	1.2 ± 0.1	5.80 ± 0.01
	500	2.76 ± 0.01 *	1.2 ± 0.3	6.02 ± 0.01 *

LA indicates atrial myosin; LV indicates ventricular myosin; V_max_ is the maximum sliding velocity of thin filaments at a saturating Ca^2+^ concentration; *h* is the Hill cooperativity coefficient; and pCa_50_ is the Ca^2+^ concentration at which the velocity is half-maximal and the Ca^2+^ sensitivity. Simbol * indicates differences between the values of the parameters of the Hill equation with and without rat Tmod1 (Mann–Whitney U test, *p* < 0.05).

**Table 4:** The myosin concentration required to achieve the half-maximal filament velocity (C_myosin_).

Myosin	Filament	Tmod Concentration (nM)	C_myosin_ (µg/mL)
Sheep LV	F-actin–Tpm1.1 filament	0 nM Tmod1	45.7 ± 5.2
		500 nM Tmod1	15.0 ± 2.2 *
Sheep LV	F-actin–Tpm1.2 filament	0 nM Tmod1	35.5 ± 5.1
		500 nM Tmod1	27.4 ± 6.7
Sheep LV	pCa 4, thin filament containing Tpm1.1	0 nM Tmod4	38.6 ± 4.1
		500 nM Tmod4	33.5 ± 5.2
Sheep LA	pCa 4, thin filament containing Tpm1.1	0 nM Tmod1	56.4 ± 4.3
		500 nM Tmod1	37.9 ± 15.3
Sheep LA	pCa 4, thin filament containing Tpm1.2	0 nM Tmod1	38.7 ± 3.1
		500 nM Tmod1	39.3 ± 5.3
Sheep LV	pCa 5.2, thin filament containing Tpm1.1	0 nM Tmod1	27.9 ± 2.3
		500 nM Tmod1	33.3 ± 5.1

LA indicates atrial myosin; LV indicates ventricular myosin; and C_myosin_ is the myosin concentration required to achieve the half-maximal filament velocity. Simbol * indicates differences between the values of the parameters of the Hill equation with and without rat Tmod (Mann–Whitney U test, *p* < 0.05).

**Table 5:** Peak areas and elution volumes obtained from size-exclusion chromatography profiles of Tmod1.

SEC Profile Name	Peak Number					
	Peak 1		Peak 2		Peak 3	
	Peak Area (ml * abs)	Elution Volume (ml)	Peak Area (ml * abs)	Elution Volume (ml)	Peak Area (ml * abs)	Elution Volume (ml)
Tpm1.1	21.19	12.9	-	-	-	-
Tpm1.2	14.89	13.0	-	-	-	-
Tmod1	28.97	16.7	-	-	-	-
Tpm1.1 and Tmod1	25.87	12.6	15.18	16.7	-	-
Tpm1.2 and Tmod1	25.37	12.6	1.30	15.8	14.46	16.7

**Table 6:** Peak areas and elution volumes obtained from size-exclusion chromatography profiles of Tmod4.

SEC Profile Name	Peak Number					
	Peak 1		Peak 2		Peak 3	
	Peak Area (ml * abs)	Elution Volume (ml)	Peak Area (ml * abs)	Elution Volume (ml)	Peak Area (ml * abs)	Elution Volume (ml)
Tpm1.1	17.59	12.9	-	-	-	-
Tpm1.2	12.36	13.0	-	-	-	-
Tmod4	9.45	16.7	-	-	-	-
Tpm1.1 and Tmod4	17.67	12.8	3.15	16.5	-	-
Tpm1.2 and Tmod4	17.10	12.9	1.23	15.7	1.64	16.5

## Data Availability

All data are presented in this article.

## Supplementary Materials

The following supporting information can be downloaded at https://www.mdpi.com/article/10.3390/biom15050727/s1, Figure S1: Gel electrophoresis of heavy chain isoforms of sheep and rat cardiac myosin; Figure S2: The effect of Tmod on filament length. (A) F-actin, thin filaments reconstructed from troponin and Tpm1.1, and native thin filaments (NTFs) on a myosin-coated surface in a flow cell. (B) Filament length; Figure S3: Sequence alignment of Tpm1.1 and Tpm1.2 from Homo sapiens; Figure S4: Alignment of Tpm-binding sites 1 (amino acids 1–39) and 2 (amino acids 109–145) of Tmod1 and Tmod4 from Homo sapiens; Table S1: Effect of Tmod on filament length.

## Abbreviations

The following abbreviations are used in this manuscript:

LV	left ventricle;
LA	left atria;
SEC	size-exclusion chromatography.
