# Supplementary material for: Tropomodulin–Tropomyosin Interplay Modulates Interaction Between Cardiac Myosin and Thin Filaments
Source: Biomolecules. 2025 May 16;15(5):727. doi: 10.3390/biom15050727 (PMC12109978; doi:10.3390/biom15050727)
Supplement: Supplementary file 1 [file biomolecules-15-00727-s001.zip › biomolecules-3614049 - original images.pdf]

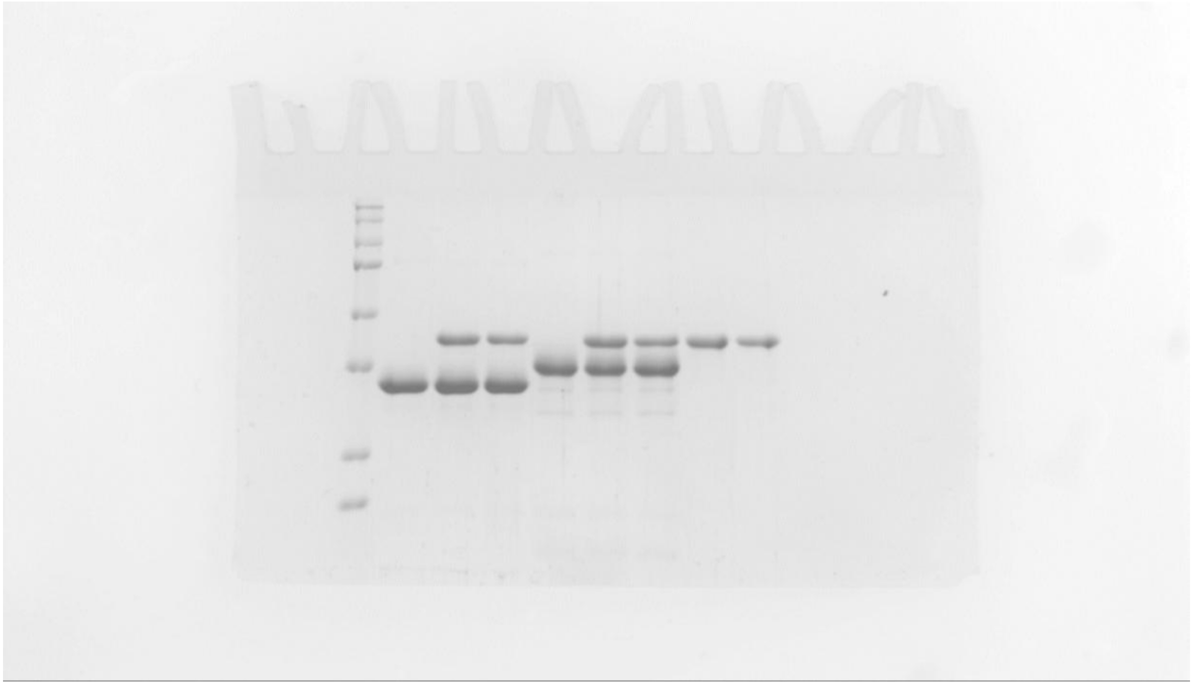

This figure represents Figure 9A in the main text.

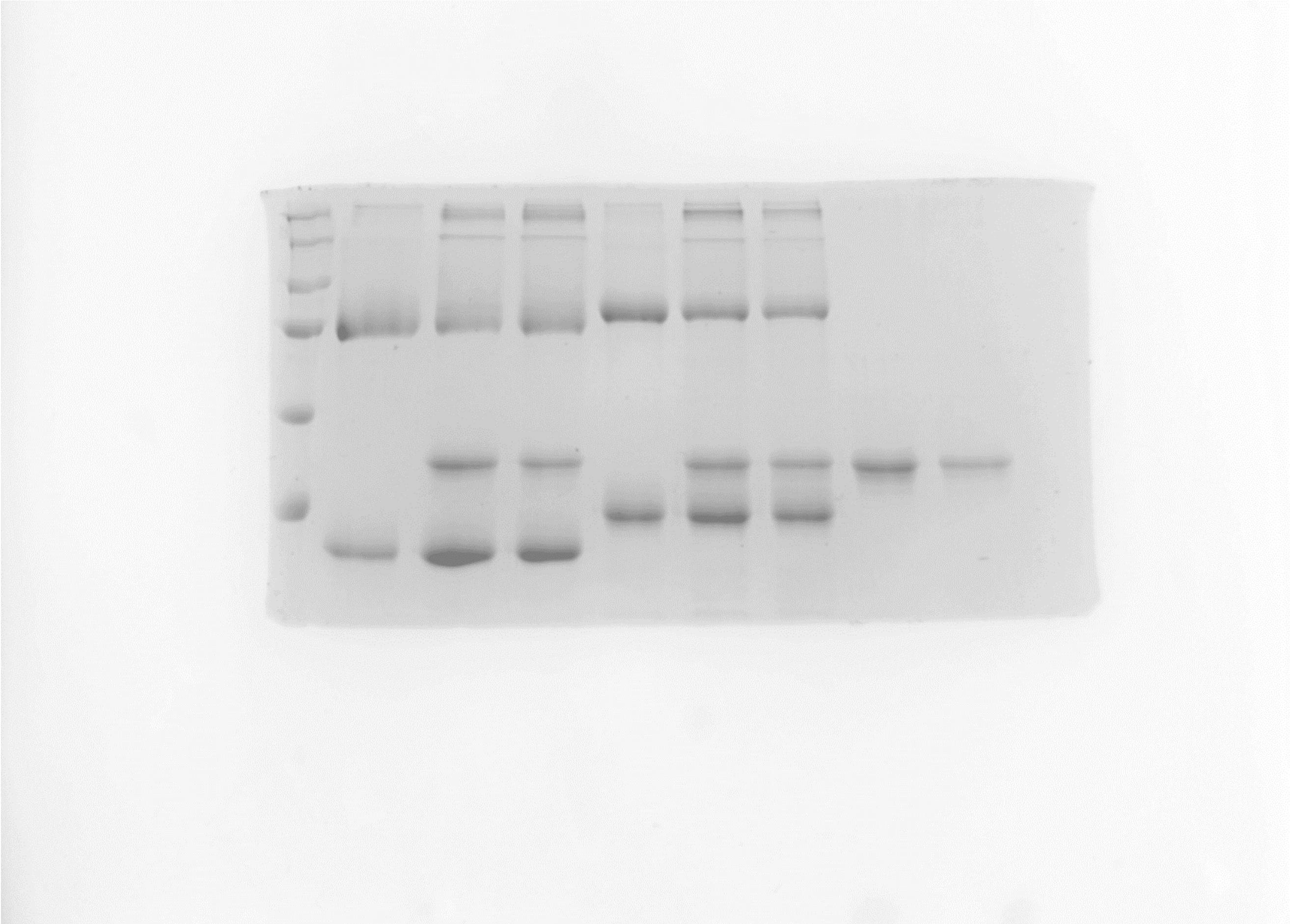

This figure represents Figure 9B in the main text.

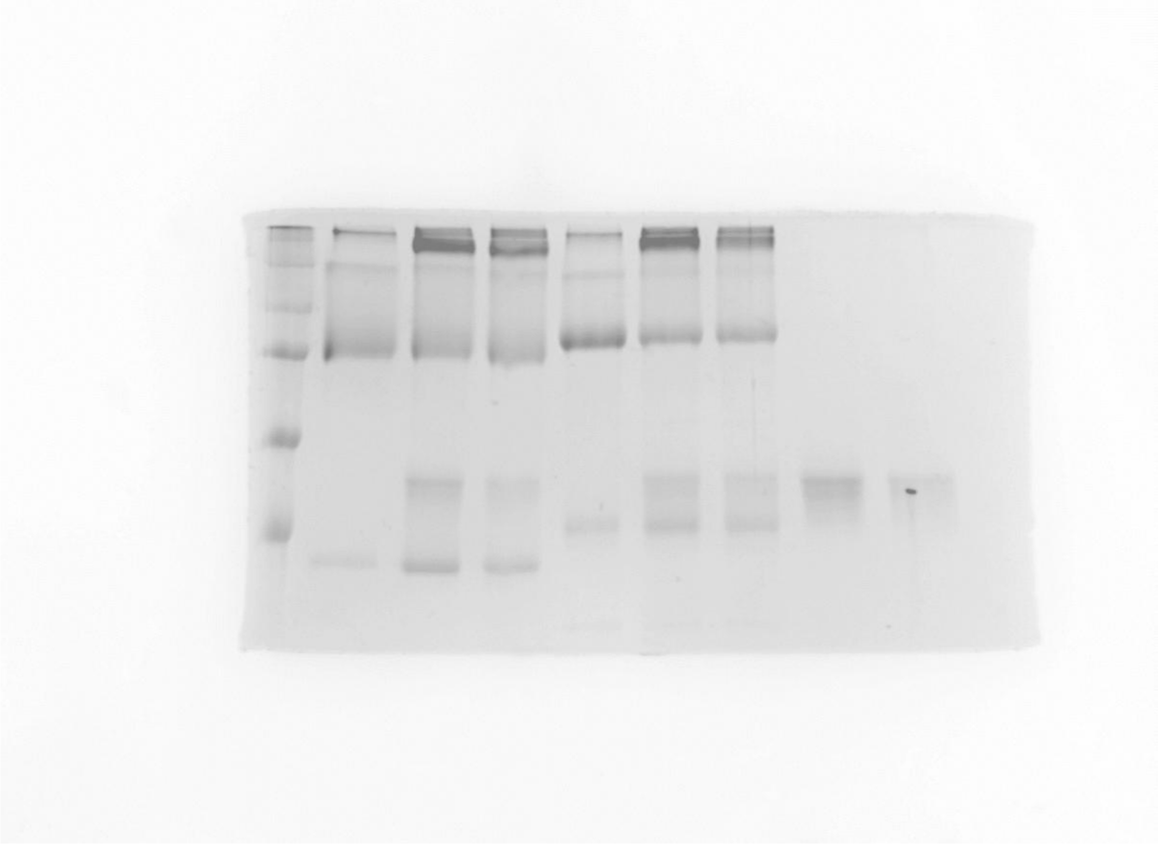

This figure represents Figure 9C in the main text.
