# Supplementary material for: Tropomodulin–Tropomyosin Interplay Modulates Interaction Between Cardiac Myosin and Thin Filaments
Source: Biomolecules. 2025 May 16;15(5):727. doi: 10.3390/biom15050727 (PMC12109978; doi:10.3390/biom15050727)
Supplement: Supplementary file 1 [file biomolecules-15-00727-s001.zip › biomolecules-3614049-supplementary.pdf]

1. Isoform composition of sheep and rat cardiac myosin

The isoform composition of myosin heavy chain (MHC) was determined by SDS-PAGE with SYPRO Ruby (Thermo Fisher Scientific, United States) staining according to [P.J. Reiser, W.O. Kline, Electrophoretic separation and quantitation of cardiac myosin heavy chain isoforms in eight mammalian species, Am. J. Physiol. 274(3) Pt 2) (1998) H1048-53.]. To determine the ratio of the MHC isoforms, the gel was imaged on a ChemiDoc MP Imaging System (Bio-Rad, Hercules, CA, United States) and band densities were determined with Image Lab 5.2.1 software (Bio-Rad, Hercules, CA, United States).

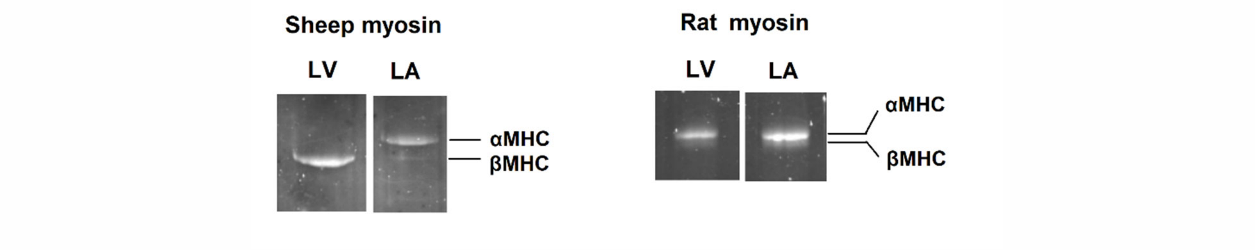

Figure S1. Gel electrophoresis of the heavy chain isoforms of sheep and rat cardiac myosin. SYPRO Ruby staining. The myosin from the sheep left atria (LA) contained 80±8% αMHC and 20±8% βMHC; the myosin from the sheep left ventricles (LVs) contained 100% βMHC. The myosin from the rat LA contained 90±6% αMHC and 10±6% βMHC; the myosin from the sheep LVs contained 90±5% βMHC and 10±5% βMHC.

2. Effect of Tmod on filament length

Tropomodulin did not affect F-actin or thin filament length (Figure S2 and Table S1).

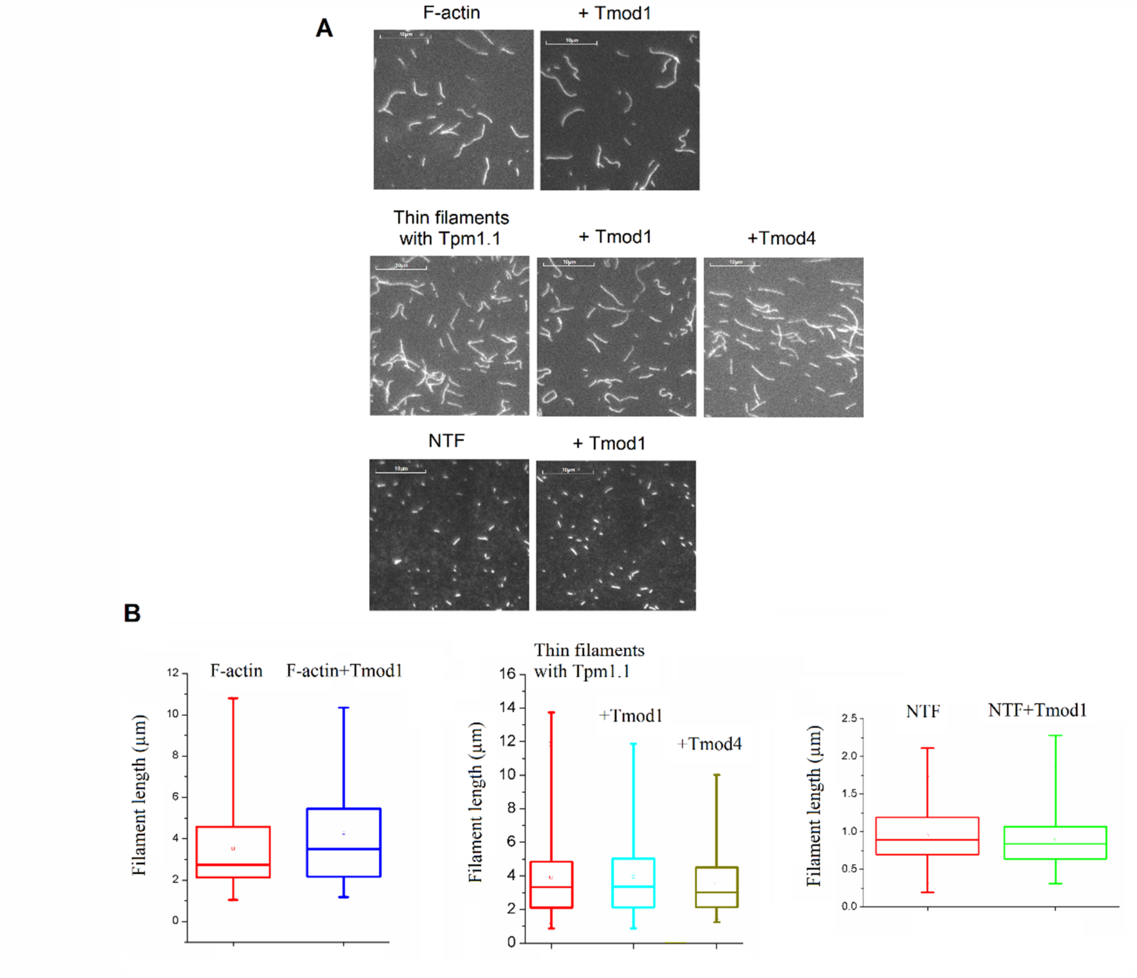

Figure S2. The effect of Tmod on filament length. (A) F-actin, thin filaments reconstructed from troponin and Tpm1.1, and native thin filaments (NTFs) on a myosin-coated surface in a flow cell. (B) Filament length. The data are presented as medians with interquartile ranges (Q1–Q3).

Table S1. Effect of Tmod on filament length

Filament	Filament length, µm		
	0 nM Tmod	500 nM Tmod1	500 nM Tmod4
F-actin	2.7 (1.0-4.6; N=55)	3.6 (1.2-5.8: N=48)	
Thin filament with Tpm1.1	3.3 (0.9-4.9; N=132)	3.4 (0.9-5.1; N=149)	3.0 (1.3-4.5; N=130)
NTF	0.89 (0.19-1.19; N=107)	0.85 (0.31-1.08; N=130)	

The data are presented as medians. Minimum and maximum values are indicated in brackets. N is the number of measured filaments. The lengths of the filaments were measured in 2-3 fields of view of the microscope in 2-3 flow cells. Comparisons were made by the Mann–Whitney U test (p<0.05).

3. Alignment of Tpm1.1 and Tpm1.2 from Homo sapiens

Aligned sequences of Tpm1.1 (P09493-1) and Tpm1.2 (P09493-6) for Homo sapiens were downloaded from UniProt. The homology of Tpm1.1 and Tpm1.2 is 90.4%.

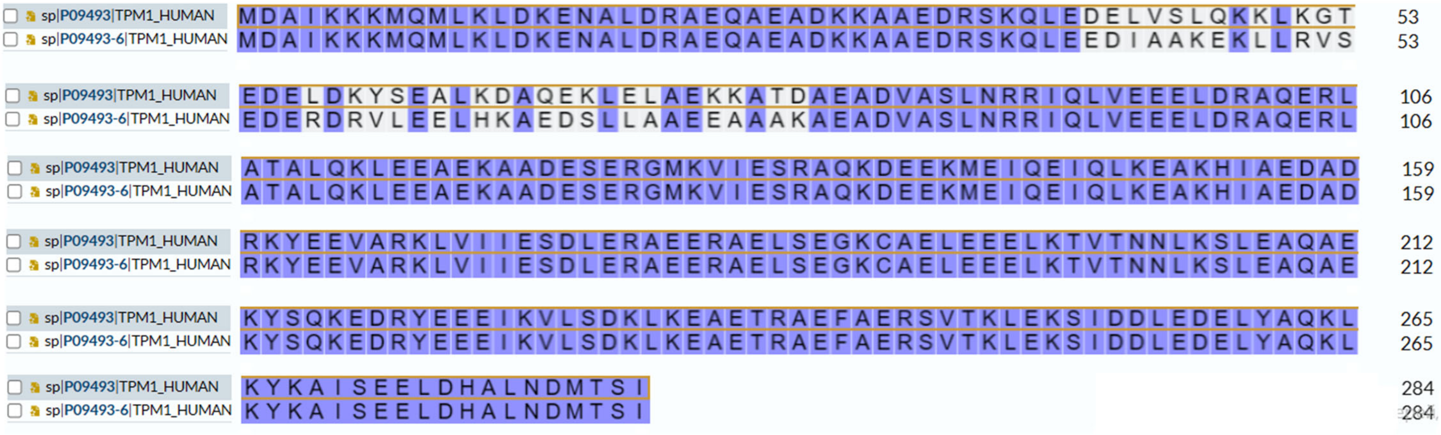

Figure S3. Sequence alignment of Tpm1.1 and Tpm1.2 from Homo sapiens.

Alignment of Tpm-binding sites 1 and 2 for Tmod1 and Tmod4 from Homo sapiens

Sequences of Tmod1 (NP_001159588.1) and Tmod4 (NP_037485.2) for Homo sapiens were downloaded from NCBI. Sequences KAI2553286.1 and KAI4082564.1 from the NCBI bank were aligned in the Mega11.06 program using the Muscle algorithm. The homology of site 1 is 61.5%, and the homology of site 2 is 78.4%.

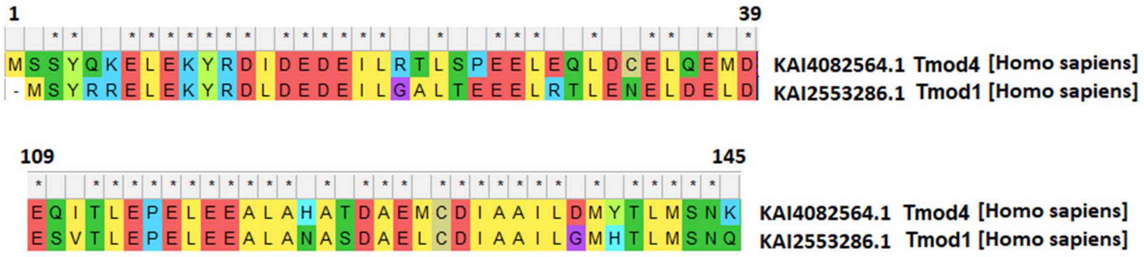

Figure S4. Alignment of Tpm-binding sites 1 (amino acids 1-39) and 2 (amino acids 109-145) of Tmod1 and Tmod4 from Homo sapiens.
