# Supplementary material for: Tropomodulin–Tropomyosin Interplay Modulates Interaction Between Cardiac Myosin and Thin Filaments
Source: Biomolecules. 2025 May 16;15(5):727. doi: 10.3390/biom15050727 (PMC12109978; doi:10.3390/biom15050727)
Supplement: Supplementary file 1 [file biomolecules-15-00727-s001.zip › Original gels_Suppl.pdf]

**Gel electrophoresis of sheep myosin heavy chain.** Fragment of this gel corresponds to Figire S1 (Supplementary material). The fragment is highlighted with a rectangle. (LV left ventricle, LA – left atrium)

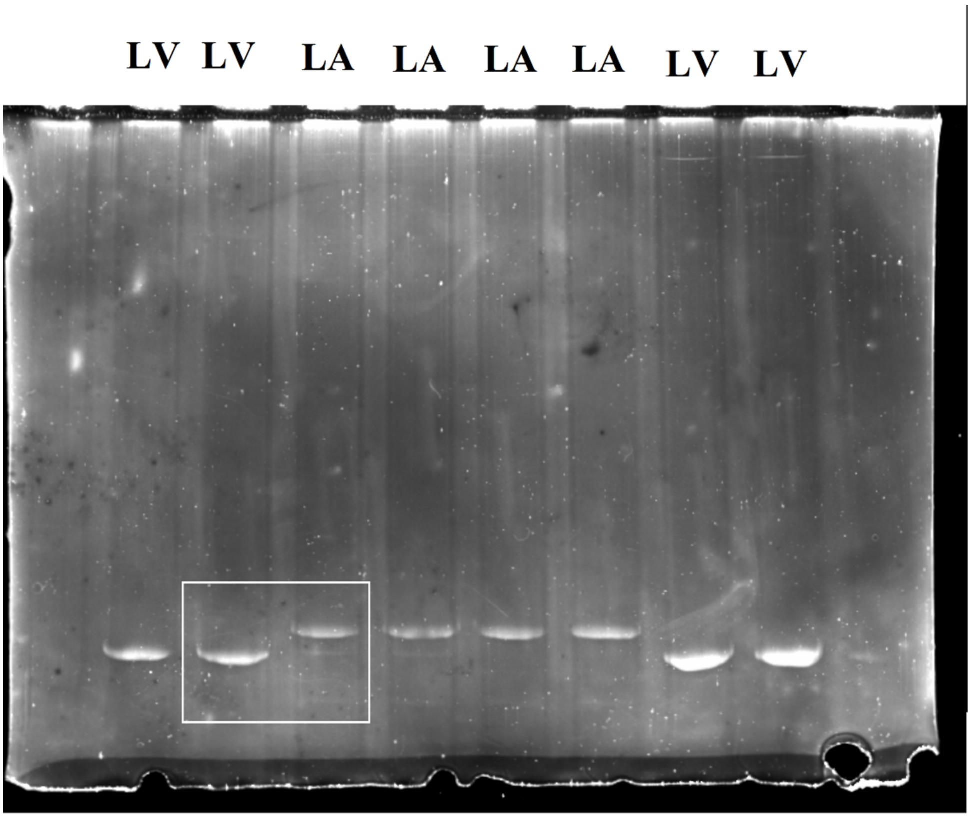


**Gel electrophoresis of rat myosin heavy chain.** Fragment of this gel corresponds to Figire S1 (Supplementary material). The fragment is highlighted with a rectangle. (LV left ventricle, LA – left atrium)

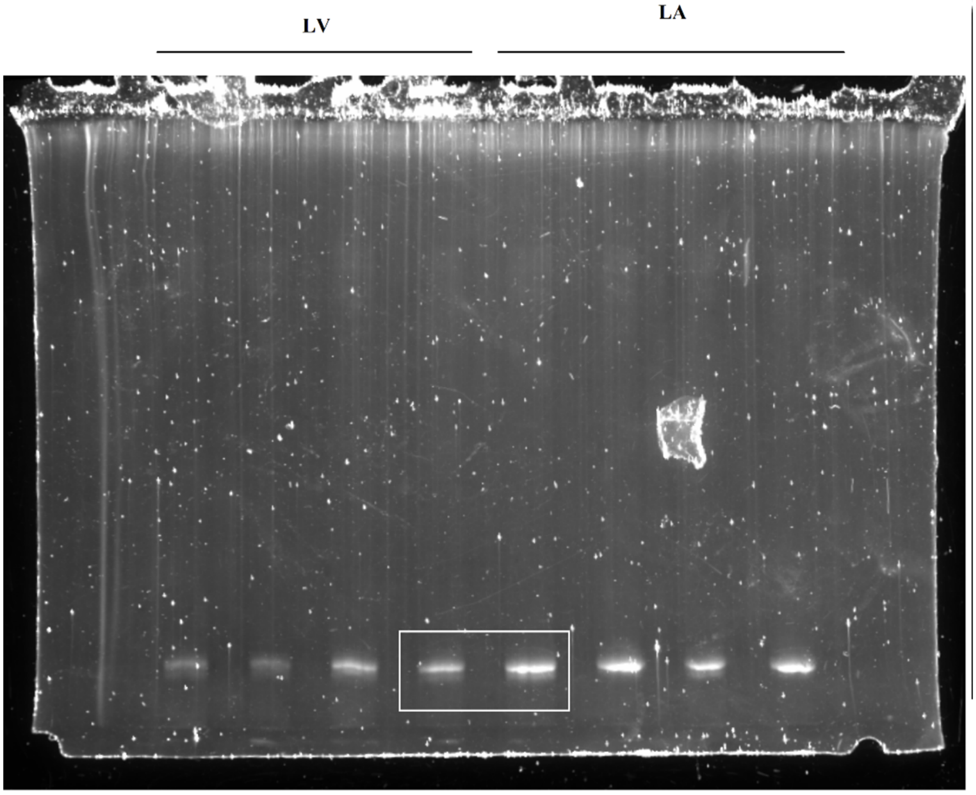
LV
LA
